# Comparison of 0.1%, 0.18%, and 0.3% Hyaluronic Acid Eye Drops in the Treatment of Experimental Dry Eye

**DOI:** 10.1089/jop.2018.0032

**Published:** 2018-10-11

**Authors:** In Cheon You, Ying Li, Rujun Jin, Min Ahn, Won Choi, Kyung Chul Yoon

**Affiliations:** ^1^ Department of Ophthalmology, Research Institute of Clinical Medicine of Chonbuk National University-Biomedical Research Institute of Chonbuk National University Hospital, Jeonju, Korea.; ^2^ Department of Ophthalmology and Research Institute of Medical Sciences, Chonnam National University Medical School and Hospital, Gwangju, Korea.

**Keywords:** dry eye disease, 0.1% hyaluronic acid, 0.18% hyaluronic acid, 0.3% hyaluronic acid

## Abstract

***Purpose:*** To compare the efficacy of 0.1%, 0.18%, and 0.3% hyaluronic acid (HA) artificial tear in the treatment of experimental dry eye (EDE).

***Methods:*** EDE was established in female C57BL/6 mice through an air draft and subcutaneous scopolamine injection. The mice were divided into 5 groups according to topical treatment regimens (*n* = 5 each): EDE control, balanced salt solution (BSS), preservative-free 0.1% HA, 0.18% HA, and 0.3% HA. The tear film break-up time (TBUT) and corneal fluorescein staining scores were measured 5, 10, 14, 21, and 28 days after treatment. The corneal smoothness scores were measured. In addition, periodic acid–Schiff (PAS) and terminal deoxynucleotidyl transferase dUTP nick end labeling (TUNEL) staining were performed.

***Results:*** The values for TBUT and corneal fluorescein staining showed greater improvements in all the HA groups (*P* < 0.05) than in the EDE and BSS groups after 10 days of treatment. Mice treated with 0.3% HA showed a more significant improvement in all clinical parameters than did those in the EDE control, BSS, 0.1% HA, and 0.18% HA groups (all *P* < 0.05) after 28 days of treatment. The goblet cell counts were higher in the 0.3% and 0.18% HA groups than in the 0.1% HA group. The number of TUNEL-positive cells was the lowest in the 0.3% HA group.

***Conclusions:*** In EDE, 0.3% HA artificial tears are more effective than the 0.1% and 0.18% HA in improving tear film instability and ocular surface staining and irregularity, in increasing the number of conjunctival goblet cells, and in decreasing corneal epithelial apoptosis.

## Introduction

Dry eye disease (DED) is a multifactorial disease of the tears and ocular surface that is characterized by a loss of homeostasis of the tear film, and accompanied by ocular symptoms.^1^ The tear film instability and hyperosmolarity, ocular surface inflammation and damage, and neurosensory abnormalities are etiologic causes associated with DED.^1^ The prevalence of DED ranged from 5% to 50%.^2^ Conventionally, DED is characterized by aqueous deficiency of tear volume loss, early tear film breakup, and increased evaporative loss from the ocular surface.^3^ The tear film is composed of many substances, including lipids, proteins, mucins, and electrolytes.^3^ All of these contribute to the integrity of the ocular tear film. The central mechanism of DED is lacrimal deficiency and increased evaporative loss leading to hyperosmolar tissue damage.^4^ Research in humans and animal models has shown that this, either directly or by inducing inflammation, causes a loss of both corneal epithelial and conjunctival goblet cells.^4^ The consequent decrease in ocular surface wettability leads to more rapid tear film breakup and amplifies tear hyperosmolarity and completes the vicious circle of events that lead to ocular surface damage.^4^ The current management options for DED include treatments for tear insufficiency and eyelid abnormalities, as well as anti-inflammatory medications, surgical approaches, dietary modifications, environmental considerations, and complementary therapies.^5^ Artificial tears are traditionally the most commonly used DED therapeutic agents.^6^ These topical products are often comprised of a physiological saline with a variety of viscosity-enhancing surface lubricants formulated to replace and/or supplement the natural tear film.^6^

The preocular tear film is composed of 3 layers: an outer tear film lipid layer produced by the Meibomian glands in the tarsal plate, a central tear film aqueous layer produced by both the main and accessory lacrimal glands, and an inner tear film mucin layer produced by the goblet cells in the conjunctiva.^7,8^ Recently, it is also commonly considered that the aqueous and mucin layers are a single layer of mucoaqueous gel.^3,9^

Hyaluronic acid (HA) is an anionic glycosaminoglycan with a viscoelastic rheology.^10^ While it is found mainly in connective tissue, it is also highly concentrated in the vitreous humor and in the aqueous humor, where it coats the corneal endothelium. HA has gained widespread application in the lubricants used for treating DED because it effectively binds water, resists dehydration, and shows excellent biocompatibility.^10,11^ Previous studies have shown that HA protects corneal epithelial cells against damage, stimulates epithelial migration, and improves the optical quality of the retinal image.^10,12^ HA was effective in protecting the ocular surface from dehydration or tear film instability in porcine, rabbit, and murine dry eye models.^13–15^ HA artificial tear eye drops were also shown to improve ocular surface irregularity, stabilize precorneal tear film, and ameliorate the intensity of dry eye symptoms.^16,17^ Although traditional formulations contain HA at 0.1% concentration, other HA formulations with higher concentrations have recently been introduced.

The purpose of this study was to compare the efficacy of commercially available preservative-free 0.1% HA, 0.18% HA, and 0.3% HA artificial tear eye drops in a mouse model of environmental and pharmacological desiccating stress-induced experimental dry eye (EDE) by evaluating the changes in tear film break-up, corneal surface smoothness and corneal epithelial staining, conjunctival goblet cell density, and corneal epithelial apoptosis.

## Methods

This research protocol was approved by the Chonnam National University Medical School Research Institutional Animal Care and Use Committee. All animals were treated and all *in vivo* experiments were performed according to the institutional guidelines and the ARVO statement for the Use of Animals in Ophthalmic and Vision Research.

### Animals and agents

Female C57BL/6 mice 8 weeks of age were used in the following experiments. EDE was induced pharmacologically using a subcutaneous injection of 0.5 mg/0.2 mL scopolamine hydrobromide (Sigma-Aldrich, St. Louis, MO) 4 times a day, with an experimental desiccating environment that was created with exposure to an air draft [by placing the mice between 2 fans to obtain a continuous air flow (15 L/min)] in a 25°C room with 30% ambient humidity, as previously described.^18–22^ During these experiments, the animals' behavior and food and water intake were not restricted.

Commercially available 3 artificial tear eye drops were used. Animals in the HA groups received 0.1% preservative-free HA ophthalmic solution (0.1% Hyalein Mini^®^; Santen Pharmaceutical Co., Ltd., Osaka, Japan), 0.18% preservative-free HA ophthalmic solution (Kynex 2^®^; Alcon Korea, Seoul, Korea), or 0.3% preservative-free HA ophthalmic solution (0.3% Hyalein Mini; Santen Pharmaceutical Co., Ltd.).

### Experimental design

The mice were divided into 5 groups with random allocation sequence according to the topical treatment regimens. Five groups were as follows: EDE controls (received no eye drops), EDE+balanced salt solution (BSS), EDE +0.1% HA, EDE +0.18% HA, and EDE +0.3% HA. All mice of treatment groups were applied topically 2 μL of eye drops 4 times a day. The tear film break-up time (TBUT) and corneal fluorescein staining score were measured at 5, 10, 14, 21, and 28 days of EDE. The corneal smoothness scores were measured at 28 treatment days. Twenty-eight days after commencing the treatment, the mice were euthanized. Thereafter, histological analysis using periodic acid–Schiff (PAS) staining and terminal deoxynucleotidyl transferase dUTP nick end labeling (TUNEL) staining was performed. Experiments were repeated 3 times, with a total of 5 mice in each group.

### Evaluation of TBUT and corneal fluorescein staining score

The TBUT and corneal fluorescein staining score measurements were performed using slit lamp biomicroscopy (BQ-900; Haag-Streit, Bern, Switzerland) fitted with a cobalt blue filter. After instilling 1% sodium fluorescein (1 μL volume) into the inferior conjunctival sac using a micropipette, the eyelids were manually blinked several times to distribute the ocular tear film. The eye was held open, and the time until tear film break-up (1 or more black spots or streaks) appeared in the precorneal area was recorded with stopwatch. TBUT was measured 3 times, and the mean value was calculated in seconds. The time interval between the last instillation of study drug (artificial tear) and the evaluation of tear film break up time is at least one hour. Ninety seconds later, corneal punctate staining spots were counted in a masked fashion. Each cornea was divided into 4 quadrants that were scored individually. The corneal fluorescein staining score was calculated using a 4-point scale in each quadrant, as previously described.^23^ The scores of the 4 quadrants were summed to generate a final staining score, ranging from 0 to 16 points.

### Evaluation of corneal smoothness score

The corneal smoothness score measurements were conducted using the stereoscopic zoom microscope (SMZ 1500; Nikon, Tokyo, Japan). Without anesthesia, reflected images of a white ring from the fiber optic ring illuminator of the microscope on the corneal epithelium were taken to digital images. Corneal smoothness was evaluated by grading the distortion of a white ring. The corneal smoothness score was calculated using a 5-point scale based on the number of distorted quarters in the reflected ring, as previously described.^21^ (0, no distortion; 1, distortion of 1 quarter; 2, distortion of 2 quarters; 3, distortion of 3 quarters; 4, distortion of 4 quarters; 5, severe distortion with no ring recognized).

### Histological analysis

For PAS staining, conjunctival tissue was fixed in 4% paraformaldehyde overnight and embedded in paraffin. Serial sections, 6-μm thick, were cut from each sample. The sections were deparaffinized and stained with the 0.5% PAS for identification of goblet cells. Sections from each group were photographed with a microscope equipped with a digital camera. The number of positively stained goblet cells in the superior and inferior conjunctiva was counted in 3 sections from each eye by using image analysis software (Media Cybernetics, Silver Spring, MD). Data are presented as the average number of goblet cells per millimeter.

### TUNEL staining

A TUNEL assay was a method for detecting DNA fragmentation by labeling the 3′-hydroxyl termini in the double-stranded DNA breaks generated during the apoptotic cascade, widely used to identify and quantify apoptotic cells. After tissue preparation with paraffin-embedded and paraformaldehyde-fixed material, staining was performed using a commercially available kit (DeadEnd Fluorometric TUNEL System; Promega, Madison, WI) according to the manufacturer's protocols with modifications.^24,25^ The nuclei were visualized with 4′,6-diamidino-2-phenylindole (DAPI) present in the ProLong Gold Antifade Mounting Medium (Invitrogen, Carlsbad, CA). Digital images of representative areas were captured with a Leica laser scanning confocal microscope (Leica Microsystems, Heidelberg, Germany). TUNEL-positive cells in corneal epithelium were counted for the corneal cross-section. The results were expressed by averaging the TUNEL-positive cells from 3 sections per eye.

### Statistical analysis

Statistical analysis was performed using SPSS Statistics for Windows, version 18.0 (SPSS, Inc., Chicago, IL). Results are presented as mean ± standard deviation. Statistical differences between the groups were determined using a repeated-measure analysis of variance with Dunnett's *post hoc* analysis. A *P* value <0.05 was considered statistically significant.

## Results

### TBUT and corneal fluorescent staining scores

**FIG. 1.**
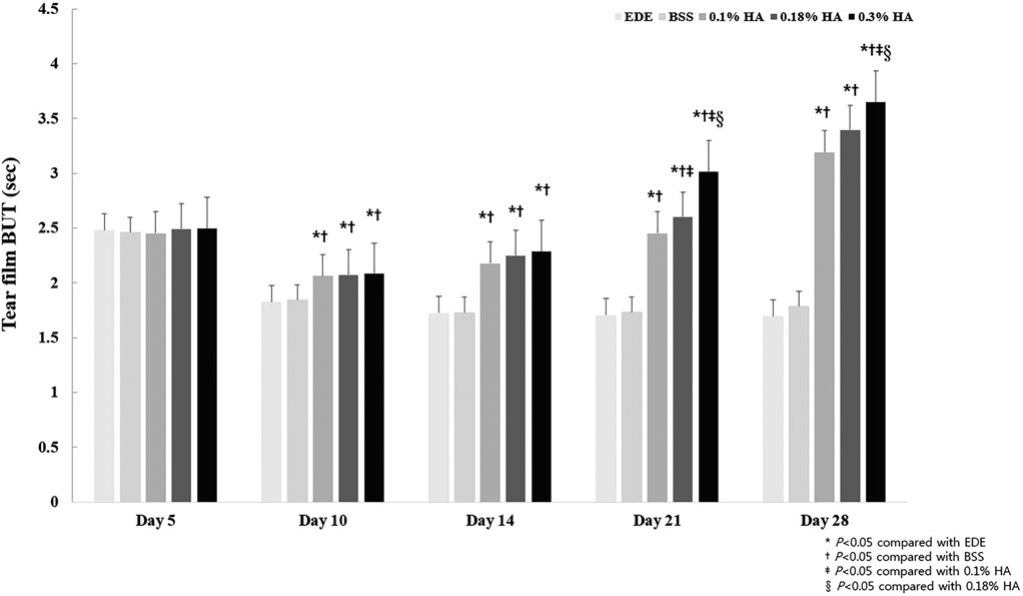
Mean TBUT changes in the EDE, 0.1% HA-treated, 0.18% HA-treated, and 0.3% HA-treated groups at days 5, 10, 14, 21, and 28. *P < 0.05 compared with the EDE group, ^†^*P* < 0.05 compared with the BSS group, ^‡^*P* < 0.05 compared with the 0.1% HA group, and ^§^*P* < 0.05 compared with the 0.18% HA group. BSS, balanced salt solution; EDE, experimental dry eye; HA, hyaluronic acid; TBUT, tear film break-up time.

At 5 days of EDE, the mean TBUT was 2.48 ± 0.08 s (control), 2.46 ± 0.19 s (BSS), 2.45 ± 0.27 s (0.1% HA), 2.49 ± 0.25 s (0.18% HA), and 2.50 ± 0.17 s (0.3% HA group). Ten days after treatment, the mean TBUT showed more significant improvements in all the HA groups (all *P* < 0.01) than in the EDE control and BSS groups (Fig. 1). Twenty-one days after treatment, a significantly higher improvement in TBUT was noted in the 0.18% HA group than in the 0.1% HA group (*P* = 0.047), as well as in the 0.3% HA group than in the 0.1% and 0.18% HA groups (both *P* < 0.01). Twenty-eight days after treatment, TBUT in the 0.3% HA group was higher than that in the 0.1% and 0.18% HA groups (both *P* < 0.01).

**FIG. 2.**
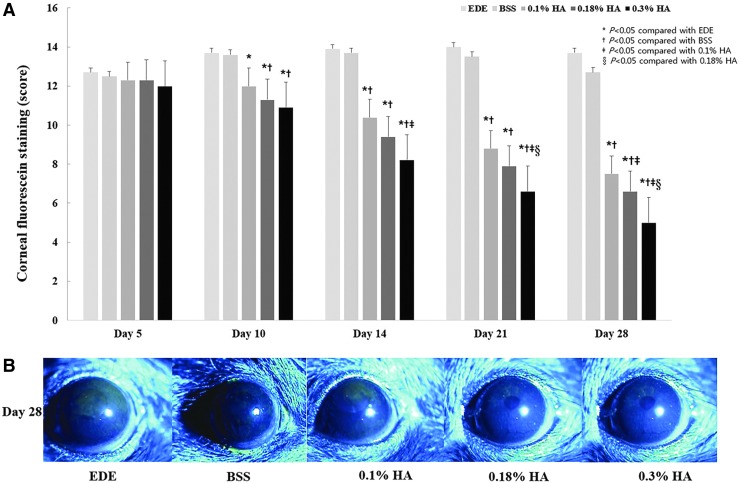
Mean corneal staining scores **(A)** and representative photographs **(B)** in the EDE, 0.1% HA-treated, 0.18% HA-treated, and 0.3% HA-treated groups at days 5, 10, 14, 21, and 28. *P < 0.05 compared with the EDE group, ^†^*P* < 0.05 compared with the BSS group, ^‡^*P* < 0.05 compared with the 0.1% HA group, and ^§^*P* < 0.05 compared with the 0.18% HA group.

At 5 days of EDE, the mean corneal fluorescein scores of the control, BSS, 0.1% HA, 0.18% HA, and 0.3% HA groups were 12.7 ± 1.49, 12.5 ± 1.27, 12.3 ± 0.68, 12.3 ± 0.95, and 12.0 ± 1.25, respectively. Ten days after treatment, the mean corneal fluorescein scores showed more significant improvements in all the HA groups (all *P* < 0.05) than in the EDE control (Fig. 2A). Twenty-one days after treatment, a significantly higher improvement in the corneal fluorescein score was observed in the 0.3% HA group than in the 0.1% and 0.18% HA groups (both *P* < 0.01). Twenty-eight days after treatment, the score in the 0.18% HA group was lower than that in the 0.1% HA group (*P* < 0.01), and the score in the 0.3% HA group was lower than that in the 0.1% HA (*P* < 0.01) and 0.18% HA (*P* = 0.02) groups. Representative biomicroscopic photographs showing the degrees of corneal staining in each group at 28 days of EDE are shown in Fig. 2B.

### Corneal smoothness score

**FIG. 3.**
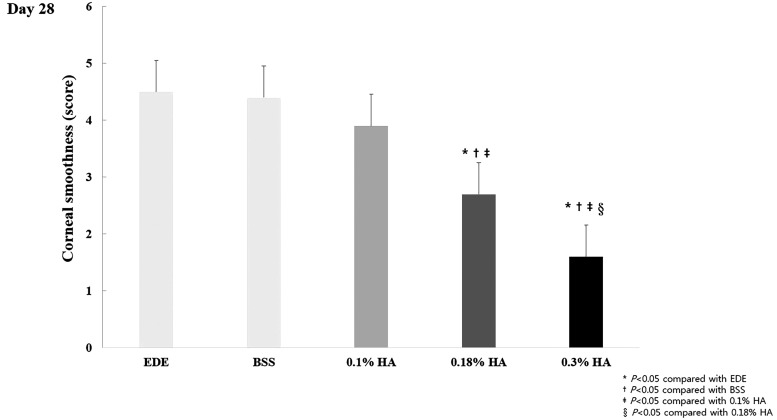
Mean corneal smoothness scores in the EDE, 0.1% HA-treated, 0.18% HA-treated, and 0.3% HA-treated groups at day 28. *P < 0.05 compared with the EDE group, ^†^*P* < 0.05 compared with the BSS group, ^‡^*P* < 0.05 compared with the 0.1% HA group, and ^§^*P* < 0.05 compared with the 0.18% HA group.

At 28 days of EDE, the mean corneal smoothness scores of the EDE control, BSS, 0.1% HA, 0.18% HA, and 0.3% HA groups were 4.5 ± 0.53, 4.4 ± 0.97, 3.9 ± 0.57, 2.7 ± 0.48, and 1.6 ± 0.70, respectively. The corneal smoothness scores had decreasing tendency with a higher concentration of HA. As for intergroup comparisons, a significantly higher improvement in the corneal smoothness score was observed in the 0.3% HA group than in the 0.1% and 0.18% HA groups (both *P* < 0.01) (Fig. 3).

### Histological analysis

**FIG. 4.**
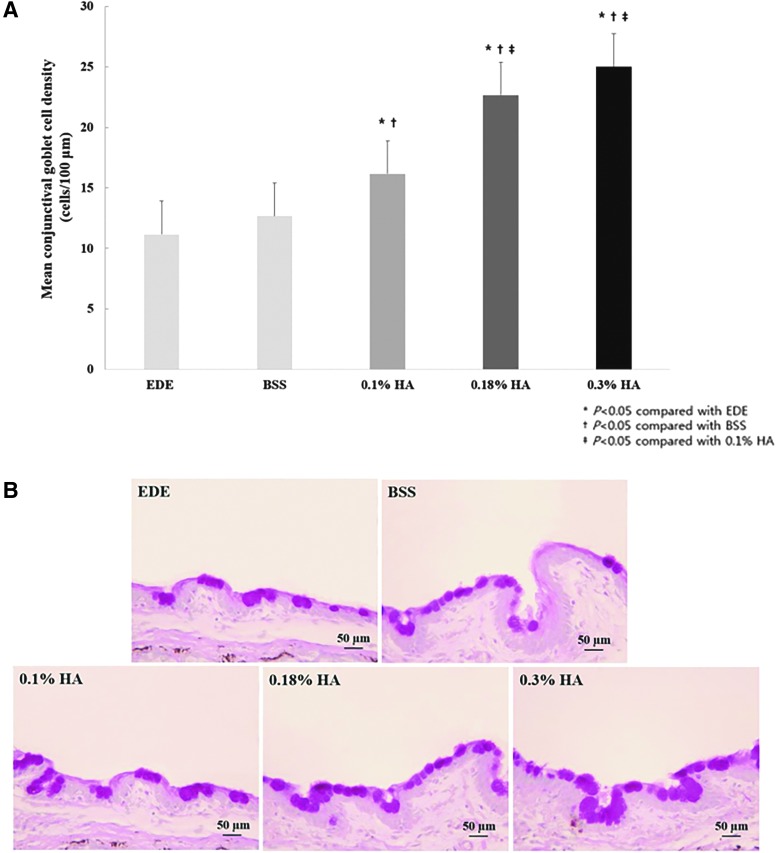
Mean number of goblet cells **(A)** and representative photographs **(B)** of Periodic acid–Schiff stains of conjunctival specimens in the EDE, 0.1% HA-treated, 0.18% HA-treated, and 0.3% HA-treated groups at day 28. *P < 0.05 compared with the EDE group, ^†^*P* < 0.05 compared with the BSS group, and ^‡^*P* < 0.05 compared with the 0.1% HA group.

The mean goblet cell counts at day 28 were 11.2 ± 0.8 cells/100 μm, 12.7 ± 1.8 cells/100 μm, 16.2 ± 1.2 cells/100 μm, 22.7 ± 1.5 cells/100 μm, and 25.0 ± 0.9 cells/100 μm in the control, BSS, 0.1% HA, 0.18% HA, and 0.3% HA groups, respectively (Fig. 4A). Goblet cell counts in all the HA groups were higher than those in the control and BSS groups (all *P* < 0.05). Both the 0.18% HA- and 0.3% HA-treated mice showed significantly higher goblet cell counts than did the 0.1% HA-treated mice (both *P* < 0.01). Representative histological findings showing the conjunctival goblet cells in each group are presented in Fig. 4B.

### TUNEL staining

**FIG. 5.**
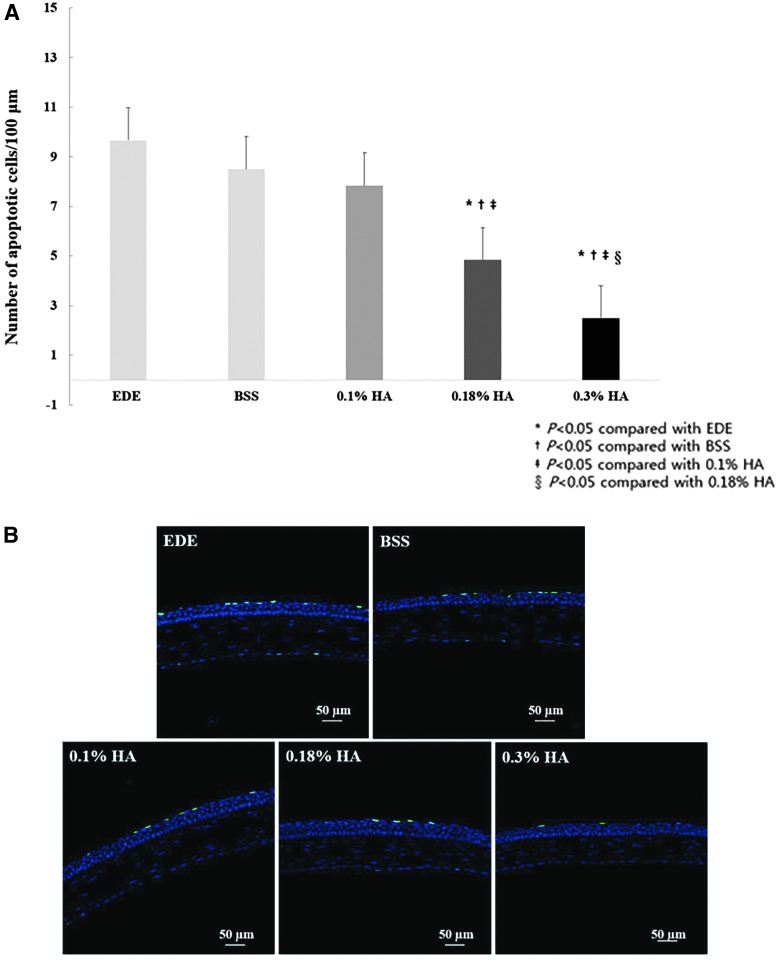
Mean number of apoptotic cells **(A)** and representative photographs **(B)** of Terminal dUTP nick end labeling assay showing the apoptotic cells in the cornea of the EDE, 0.1% HA-treated, 0.18% HA-treated, and 0.3% HA-treated groups at day 28. *P < 0.05 compared with the EDE group, ^†^*P* < 0.05 compared with the BSS group, ^‡^*P* < 0.05 compared with the 0.1% HA group, and ^§^*P* < 0.05 compared with the 0.18% HA group.

The mean apoptotic cell counts at day 28 were 9.7 ± 1.4 cells/100 μm, 8.5 ± 1.1 cells/100 μm, 7.8 ± 0.8 cells/100 μm, 4.8 ± 0.8 cells/100 μm, and 2.5 ± 1.1 cells/100 μm in the control, BSS, 0.1% HA, 0.18% HA, and 0.3% HA groups, respectively (Fig. 5A). Magnified images of the representative corneal sections stained with TUNEL (green) and counterstained with DAPI (blue) are demonstrated in Fig. 5B. EDE groups resulted in more increased apoptotic cells in the corneal epithelium than in the HA-treated groups. A significantly decreased number of TUNEL-positive cells was identified in the 0.18% HA group (all *P* < 0.01, vs. the EDE group; vs. the BSS group; vs. the 0.1% HA group) and in the 0.3% HA group (*P* < 0.01, vs. the EDE group; *P* < 0.01, vs. the BSS group; *P* < 0.01, vs. the 0.1% HA group; *P* = 0.01, vs. the 0.18% HA group).

## Discussion

HA is a naturally occurring extracellular matrix glycosaminoglycan.^26^ It has a huge capacity to bind with water and is resistant to desiccation.^27^ *In vitro* experiment showed that HA was significantly better than carboxymethylcellulose and hydroxypropyl methylcellulose at water retention and protection of corneal epithelial cells from desiccating stress.^27^ These findings demonstrated that HA could play a role not only in retaining water but also in acting as a reservoir of slowly released water molecules, which would make this ocular surface lubricant more suitable for artificial tear preparations.^27^ In humans, HA contributes to increasing tear residence time, as well as stabilizing the tear film, by maintaining a high viscosity during blinking.^10,28^ HA has also been demonstrated to promote corneal epithelial wound healing by stimulating the migration, adhesion, and proliferation of corneal epithelial cells in patients with DED.^28,29^ Administration of 0.1%, 0.15%, and 0.3% HA eye drops was effective in improving both the objective ocular surface signs and subjective symptoms in DED patients.^30^ However, in that study, the efficacy of HA eye drops did not reveal any concentration-dependent results. In the present study, we compared the efficacy of 0.1%, 0.18%, and 0.3% HA eye drops with appropriate EDE control and BSS groups.

Recently, the definition and classification subcommittee of TFOS DEWS II included “tear film instability” in their revised definition of DED.^1^ Impaired tear film stability has been a fundamental factor in diagnosing tear film abnormality, and the most frequently employed test for tear film instability is the measurement of TBUT; this is the interval of time that elapses between end of a complete blink and the appearance of the first break in the tear film.^31^ The improvements in TBUT resulting from the use of HA suggest improved integrity of the tear film, which can prevent evaporation and hyperosmolarity of the tear film owing to the rheological, mucomimetic, and water-retention properties of HA.^32^ In our study, TBUT in the higher concentration (0.3%) HA group was significantly greater than that in the 0.1% and 0.18% HA groups after experimental day 21.

In our study, the corneal fluorescein staining score, which was used for evaluating corneal surface damage, was significantly lower in the HA group than in the control group at experimental day 10; this trend was maintained until day 28. Additionally, the corneal staining score in the higher concentration 0.3% HA group was significantly lower than that in the 0.1% and 0.18% HA groups from experimental day 21 onward. This suggests that the application of higher concentration HA eye drops has a beneficial effect on the epithelial healing process of the corneal surface. Our results are in good agreement with those of previous clinical studies, which reported that HA eye drops stimulate healing of the corneal epithelium.^26,28–30,33^

DED is an immune-mediated inflammatory disease that is mediated primarily by CD4^+^ T cells.^4,34^ It is generally recognized that ocular surface inflammation and apoptosis play critical roles in the pathogenesis of DED.^4^ CD4^+^ T cells infiltrate the ocular surface after inflammatory events and secrete proinflammatory cytokines and chemokines. Specifically, IL-6 and interferon-γ are known to exacerbate the conjunctival goblet cells loss during desiccating stress.^26,35^ In previous studies, the conjunctiva of the 0.18% HA- or 0.3% HA-treated mice showed significant decreased expression of inflammatory cytokines and CD4^+^ T cell trafficking chemokines and increased conjunctival goblet cell density.^15,26^ In the present study, we found that the goblet cell density of the conjunctiva was higher in the 0.18% HA- and 0.3% HA groups than in the EDE control and 0.1% HA groups. Furthermore, the number of TUNEL-positive apoptotic corneal epithelial cells was the lowest in the 0.3% HA group. Accordingly, it can be postulated that reduced inflammation of the ocular surface due to HA artificial tears could be associated with increased goblet cell density in the conjunctiva and a decreased number of apoptotic cells in the cornea.

This study had some limitations. First, because we evaluated commercially available HA eye drops, the osmolarity was not controlled. Different treatment responses could have been achieved between isotonic and hypotonic HA eye drops in the treatment of EDE. Second, the treatment duration was relatively short. It may be insufficient to compare the effect of HA treatment on long-term DED. Third, although we evaluated the efficacy of different concentrations of artificial tear eye drops by using a murine dry eye model, the clinical response to the agents against DED in humans might vary. A larger and longer study in human is warranted to more thoroughly address corneal wound healing.

In conclusion, our study revealed that the administration of 0.1%, 0.18%, and 0.3% HA was effective in improving clinical signs, including TBUT and the corneal staining score, in EDE. Furthermore, 0.3% HA artificial tear eye drops are more effective than the 0.1% and 0.18% HA eye drops in improving tear film instability and ocular surface staining and irregularity, in increasing conjunctival goblet cell density, and in decreasing corneal epithelial apoptosis in EDE. Therefore, 0.3% HA may be the most effective among the different HA artificial tears for the treatment of DED.
